# PET Tau and Amyloid-β Burden in Mild Alzheimer’s Disease: Divergent Relationship with Age, Cognition, and Cerebrospinal Fluid Biomarkers

**DOI:** 10.3233/JAD-170129

**Published:** 2017-08-29

**Authors:** Ivan Koychev, Roger N. Gunn, Azadeh Firouzian, Jennifer Lawson, Giovanna Zamboni, Basil Ridha, Barbara J. Sahakian, James B. Rowe, Alan Thomas, Lynn Rochester, Dominic Ffytche, Robert Howard, Henrik Zetterberg, Clare MacKay, Simon Lovestone, on behalf of the Deep and Frequent Phenotyping study team ()

**Affiliations:** a Department of Psychiatry, University of Oxford, UK; b IMANOVA, Ltd; c Department of Medicine, Imperial College, UK; d NIHR Queen Square Dementia Biomedical Research Unit, University College London, London, UK; e Department of Psychiatry, University of Cambridge, Cambridge, UK; f Department of Clinical Neurosciences, University of Cambridge, UK and MRC Cognition and Brain Sciences Unit, Cambridge, UK; g Institute of Neuroscience, Newcastle University, Newcastle, UK; h King’s College London, London, UK; i Department of Molecular Neuroscience, University College London Institute of Neurology, Queen Square, London, UK; j UK Dementia Research Institute, London, UK; k Clinical Neurochemistry Laboratory, Sahlgrenska University Hospital, Mölndal, Sweden; l Department of Psychiatry and Neurochemistry, Institute of Neuroscience and Physiology, The Sahlgrenska Academy at the University of Gothenburg, Mölndal, Sweden

**Keywords:** Alzheimer’s disease, amyloid beta-peptides, cerebrospinal fluid proteins, positron emission tomography, tau proteins

## Abstract

**Background:**

Combining PET amyloid-β (Aβ) and tau imaging may be critical for tracking disease progression in Alzheimer’s disease (AD).

**Objective:**

We sought to characterize the relationship between Aβ and tau ligands as well as with other measures of pathology.

**Methods:**

We conducted a multi-center observational study in early AD (MMSE >20) participants aged 50 to 85 y. The schedule included cognitive assessments (ADAS-Cog) and CSF measurement of Aβ and tau at baseline and 6 months; PET-CT imaging with Aβ ([^18F^]AV45) and tau ([^18F^]AV1451) ligands at baseline.

**Results:**

22 participants took part in the study with 20 completing its 6-month duration and 12 having both tau and amyloid PET. The PET biomarker analysis revealed a strong negative correlation between age and tau in multiple regions. Entorhinal cortex tau and age interacted significantly in terms of cognitive change over 6 months which may have been to older participants deteriorating faster despite lower levels of cortical tau. Cortical Aβ associated with entorhinal cortex tau while CSF tau/Aβ ratio correlated strongly with cortical tau but not Aβ.

**Conclusion:**

The negative relationship between age and cortical tau whereby younger patients with mild AD had relatively greater tau burden is potentially important. It suggests that younger-age onset AD may be primarily driven by tau pathology while AD developing later may depend on a multitude of pathological mechanisms. These data also suggest that PET-tau performs better than PET-amyloid in predicting the best validated AD diagnostic marker— the CSF total tau/Aβ ratio.

## INTRODUCTION

The advent of positron emission tomography (PET) amyloid-β (Aβ) tracers [1] has made it possible to detect Aβ plaque load *in vivo*. The utility of Aβ PET is that Aβ burden predicts progression of mild cognitive impairment (MCI) to Alzheimer’s disease (AD) [2–4] and allows proof of amyloid pathology for the purposes of enrolling patients into AD clinical trials [5, 6]. However, Aβ burden is a relatively poor correlate of disease progression in established AD: *in vivo* PET measures correlate poorly with symptom severity [7–10], and its spatial distribution differs from the pattern of cortical atrophy and glucose hypometabolism [7, 8, 11, 12].

Until recently the only practical method of measuring tau pathology *in vivo* has been through CSF sampling [13, 14], which does not allow differentiation between aggregated tau pathology such as in AD and non-specific neurotoxicity. Recent advances however have led to the development of tau ligands for PET imaging [15, 16], which now enables localization of tau pathology in brain in life. One tracer, [^18^F]AV1451 (formerly called [^18^F]T807), has a binding pattern that follows closely NFTs in postmortem studies [17, 18]. The binding of the tracer is highly specific to tau relative to Aβ *in vitro* [17, 18] and has a differential uptake in AD patients relative to controls [19] and other tauopathies [20, 21]. Crucially, tau pathology measured with [^18^F]-AV1451 co-localizes with hypometabolism measured with FDG-PET [22] and is potentially useful for tracking disease progression and staging [23, 24].

In this study, we sought to clarify, early in the symptomatic phase, the relationship between the tau-selective PET tracer [^18^F]AV1451 and the more established measures of AD pathology: Aβ PET (using [^18^F]AV45 - florbetapir), CSF tau/p-tau, and Aβ, as well as cognitive performance. We recruited patients with early AD (Mini-Mental State Examination (MMSE) >20) and obtained PET, CSF, and cognition measures at the study baseline. We repeated CSF and cognitive measures at 6 months. We hypothesized that in our early AD study group, PET Aβ would correlate with tau signal in areas affected early in the disease process and that change in cognitive impairment over 6 months would be most demonstrable in the subset of patients with greater Aβ and/or tau loading. In terms of PET/CSF relationship we expected to replicate the reports of a negative correlation between PET Aβ cortical binding and CSF Aβ peptide [25, 26] and a positive relationship between CSF and PET cortical tau measures [27, 28]. Our principal aim of this analysis, though, was to determine which PET measures would best predict the most informative and currently available AD biomarker (CSF tau/Aβ ratio).

## METHODS

### Study design

A non-interventional multi-site study was carried out at the 6 centers of excellence in the UK that constitute the NIHR Translational Research Collaboration in Dementia (NIHR Biomedical Research Centers or Dementia Units associated with the following NHS Trusts: Oxford University Hospitals (OUH), South London and Maudsely (SLaM), Cambridge University Hospitals (CUHT), University College Hospital London (UCLH), West London Mental Health Care (WLMHC), and Newcastle Hospitals). Through local memory clinic services, we recruited patients aged 50–85 with a diagnosis of probable AD (with no AD pathophysiological evidence) according to National Institute of Aging-Alzheimer’s Association (NIA-AA) criteria [29]. Other inclusion criteria were i) MMSE score of 20 and above, ii) Modified Rosen-Hachinski Ischemic score of 4 or less, iii) being on stable medication dose for any non-significant medical conditions for at least one month; iv) stable dose for at least 3 months if treated with cholinesterase inhibitors and/or memantine.

We aimed to enroll 24 patients across all sites. Participants attended a screening visit where consent was obtained and eligibility assessed. This was followed by a baseline visit within 30 days, with three further assessments assessment episodes over a six-month period (baseline, month 1, month 3 and month 6). Due to the demanding nature of the first and last visits, study procedures were completed over a period of up to 5 days. The original purpose of this study was to evaluate the acceptability and feasibility of such in-depth and high frequency biomarker sampling in view of organizing a larger study following a similar design.

The full study schedule will be reported elsewhere but included demographic, clinical, and physical exam together with MMSE and assessment of sensory and motor impairment, mood and behavior, global function, and function. Cognition was assessed using pen and paper tests (Alzheimer’s Disease Assessment Scale –Cognitive subscale (ADAS-Cog) and MMSE) as well as computerized methods (Cambridge Neuropsychological Test Automated Battery (CANTAB) Paired Associates Learning (PAL), and Spatial Working Memory (SWM)).

At baseline, we performed PET for regional Aβ and tau load with all imaging being performed at a single site in London (IMANOVA Ltd). One study site was too distant for participant travel and so was not included in the PET component of the trial (Newcastle). The protocol included a T1-weighted MRI structural scan and two dynamic PET scans, one with [^18^F]AV45 (0–60 min, 150±24 MBq) and one with [^18^F]AV1451 (0–120 min, 163±10 MBq) to measure Aβ and tau respectively. The MRI scan was made on a Siemens 3T Tim Trio with a 32-channel phased array head coil. A 1 mm isotropic whole-brain structural 3D T1-weighted MPRAGE was acquired using TI = 880 ms, TR = 2000 ms and FA = 8° with a parallel imaging factor of 2 in 4 m: 54 s. For the PET scans, subjects were positioned in the PET scanner, after the insertion of a venous cannula in an antecubital or forearm vein, and a head-fixation device was used to minimize head movement during data acquisition. The PET scans were acquired on two Siemens PET/CT scanners (Hi-Rez Biograph 6 and Biograph 6 TruePoint with TrueV, Siemens Healthcare, Erlangen, Germany) while for each subject, Aβ and tau PET scans were acquired on the same scanner. A low-dose CT scan was performed immediately before each PET scan in order to estimate attenuation. A single intravenous bolus of [^18^F]AV45 (Florbetapir) tracer (150±24MBq) for a 60 min Aβ PET scan and [^18^F]AV1451 ([^18^F] T807) tracer (163±10MBq) for a 120 min tau scan was injected to each subject. The dynamic images were reconstructed using a 2D Filtered Back Projection (FBP) algorithm resulting in a 128×128 matrix with 2 mm isotropic voxels. Corrections were applied for attenuation, randoms, scatter, and tracer radioactive decay. All image processing and analysis was performed in MIAKAT™ (www.miakat.org). Dynamic PET data were corrected for motion and a neuroanatomical atlas [30] was nonlinearly registered to each subject using the individuals T1 MRI scan to enable parcellation of regions of interest. Regional standard uptake values (SUVr) with respect to cerebellar gray matter were obtained for the 30–60 min time window for [^18^F]AV45 and 60–120 min for [^18^F]AV1451 (one participant stayed in the scanner for 110 min only –we used 60–110 min time window in that instance) to investigate static outcome measures. For both tau and Aβ measures regions of interest were identified on the basis of the Braak and Braak staging model of AD [31] –entorhinal cortex (represented by gyrus ambiens) and hippocampus (stages 1-2); parahippocampus, fusiform gyrus, posterior cingulate, inferior temporal gyrus, thalamus, and amygdala (stage 3-4); frontal, parietal as well superior temporal and medial temporal cortices (stage 5-6). We also extracted overall cortical SUVr values (cortex defined as occipital, insular, temporal, frontal, cingulate, and parietal cortices) for both Aβ and tauligands.

Cerebrospinal fluid (CSF) was collected at the baseline visit and 6-month follow-up. Total tau (t-tau), phosphorylated tau (p-tau) and Aβ (i.e., Aβ_42_) concentrations were measured using INNOTEST enzyme-linked immunosorbent assays (ELISAs) (Fujirebio Europe N.V., Gent, Belgium). Well-established standard operating protocols for sample collection and management were used [32] and assays performed according to manufacturer’s instructions, as previously described in detail [33]. All measurements were performed by board-certified laboratory technicians who were blinded to clinical data. The measurements were performed on one occasion using one batch of reagents. Intra-assay coefficients of variation were below 10% and all samples measured in the quantitative range of theassays.

### Statistical analysis

SPSS (Version 22) and the RStudio statistical package (Version 0.99.896) were used to analyze the data. The relationship between Aβ and tau in the regions of interest and cortex were compared using two-way Pearson’s correlations. We followed up any significant relationships with linear models including age as a covariate to investigate its potential effects. The relationship between Aβ/tau SUVr values and age was also explored using two-way Pearson’scorrelations.

In terms of cognitive measures analyses, we used repeated measures analyses of variance (ANOVAs) on memory-based tasks where longitudinal measurements were available (ADAS-Cog, PAL, SWM) to determine whether there was a significant change to cognition over the 6-month study period. Within subject factor for each model was study visit (baseline, month 1, month 3, and month 6 for PAL and SWM; baseline and month 6 for ADAS-Cog) and outcome variables were respectively: ADAS-Cog total score, PAL total projected errors and SWM between search errors. Sex was selected as between-subject factor for the three models while age was evaluated as a covariate. We explored the effect of PET tau and Aβ SUVr levels on baseline and longitudinal cognition (calculated by subtracting baseline cognitive score from 6-month score for cognitive variable) using linear models. We repeated these analyses with entorhinal cortex tau (due to a significant correlation we found between cortical Aβ and entorhinal tau, see results section) and posterior cingulate Aβ (due to reports of posterior cingulate Aβ predicting cognitive progression [3]) as independent variables. Only ADAS-Cog scores were included in these analyses as SWM and PAL mean error variables were not normally distributed in the sample of patients who underwent PET.

We analyzed CSF concentrations for Aβ, tau, p-tau, and also generated tau/Aβ ratios. We performed two-way Pearson’s correlations between selected SUVr values (cortical tau and Aβ) and the four CSF measures at baseline visit (Aβ, tau, p-tau, and tau/Aβ ratio). To test the hypothesis that either cortical Aβ or tau may predict CSF change over the 6-month study period we ran linear models with cortical Aβ and tau as independent variables and CSF measures (tau, p-tau, Aβ, and tau/Aβ) as repeated dependent variables (baseline and 6-month follow-up). Finally, we explored the CSF markers’ association with longitudinal cognition by performing linear models with baseline (i.e., visit 1) CSF tau, p-tau, Aβ, and tau/Aβ ratio as independent variables and ADAS-Cog and PAL score change over 6 months (i.e., baseline scores subtracted from 6-month scores) as dependent variables (SWM scores were not included as they were not normally distributed in the subsample of patients who had baseline CSF). We tested the moderating effect of age in these models.

### Ethics

The study was approved by a National Research Ethics Committee London on the 19th of Sept. 2014 –IRAS reference 14/LO/1467, IRAS project ID: 156309. All participants had mental capacity for informed consent.

## RESULTS

### Recruitment

Eight participants were excluded at screening: 5 because they failed to meet study entry criteria, one because of having had a recent PET scan, one for withdrawal of consent, and one for an unspecified reason. Twenty-two participants were included after screening and 20 remained at follow-up (one drop-out due to consent withdrawal and one exclusion due to psychosis). All 20 participants completed the cognitive assessments as planned (CANTAB and paper-based tests). Out of the 20 participants, 16 had CSF testing at baseline (2 did not complete due to medical reasons and 2 due to logistical reasons). At follow-up CSF was taken from 16 participants –reasons for not doing the testing were again medical (2) and logistical (2) but this was the case for three people who had completed the baseline CSF (i.e., three participants who had follow-up CSF did not have baseline CSF). Therefore 13 participants had CSF testing both at baseline and follow-up. We planned to perform PET imaging at five out of the six study centers and 15 of the 16 eligible study completers underwent imaging (one missing imaging because of logistical reasons). PET Aβ data with [^18^F]AV45 was successfully obtained for 14 out of 15 participants (1 participant had unusable data due to excessive motion) and PET tau data with [^18^F]AV1451 for 12 of the 15 participants (3 participants did not complete at least 70 min of image acquisition).

See Table 1 for demographics of patients included in the study.

**Table 1:** Demographics

Demographics	Mean	SD
Sex	11M 9F
Age	71.3	8.4
Education (y)	14.7	3.9
Premorbid IQ	116.0	8.2
MMSE	24.8	2.4
ADAS-Cog	14.6	6.1
Rosen-Hachinski score	0.7	0.9
Clinical Dementia Rating score	0.63	0.27
Geriatric Depression Scale	2.2	1.34
Bristol Activities of Daily Living Scale	3.2	3.0

### PET tracer analyses

**Fig.1:**
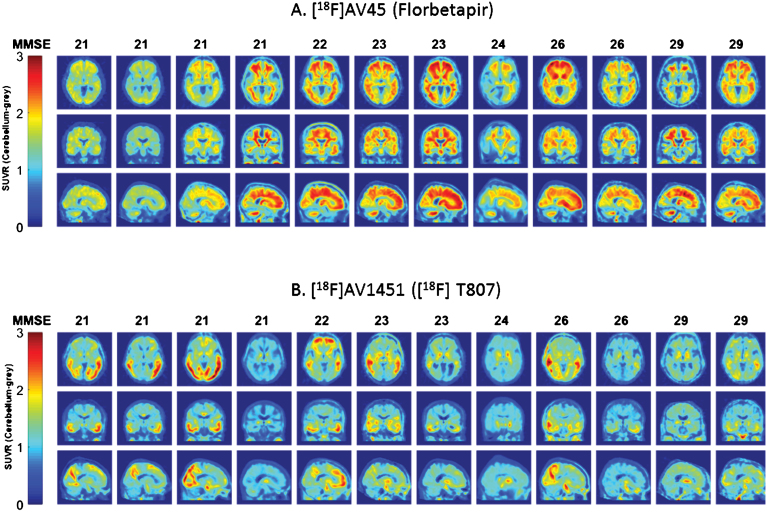
Topographical plots of PET SUVr levels for the 12 participants who underwent both Aβ and tau imaging. A and B demonstrate, respectively, [^18^F]AV45 (Aβ) and [^18^F]AV1451 (tau) topographical distribution of SUVr signal across the 12 participants (each column represents a participant; participants have been ordered according to baseline MMSE scores).

**Fig.2:**
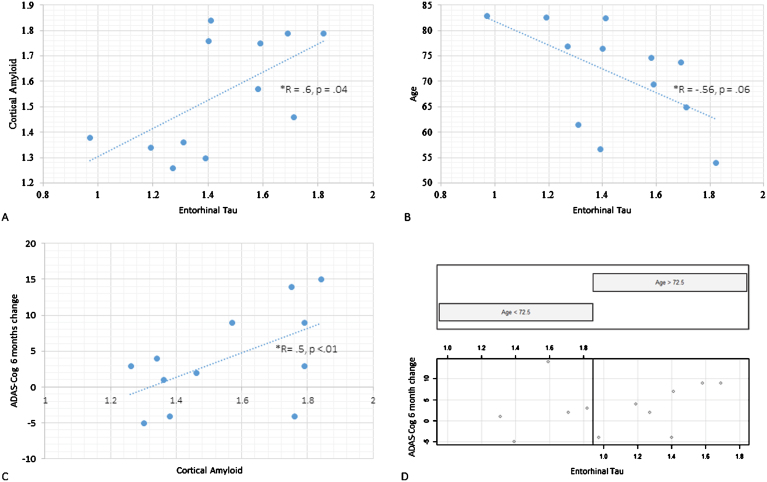
PET tau and Aβ scatterplots: A) Cortical Aβ (vertical axis) and entorhinal tau (horizontal axis) scatterplot. B) Age (vertical axis) and entorhinal (horizontal axis) tau scatterplot. C) Illustration of the interaction between age and entorhinal tau on cognitive progression. ADAS-Cog score change from baseline to 6-month follow-up on the vertical axis, entorhinal tau on the horizontal axis. Scatterplot is split with left side featuring those under the median age (72.5 y) and the right with those above it. D) ADAS-Cog score change from baseline to 6-month follow-up (vertical axis) and cortical Aβ (horizontal axis).

Our regional correlation analysis (12 participants included) revealed that cortical Aβ was associated with tau in the entorhinal cortex (*r* = 0.6, *p* = 0.04, Fig. 2A), thalamus (*r* = 0.72, *p* < 0.01) and parahippocampus (*r* = 0.63, *p* = 0.03). This relationship was not moderated by age; see Fig. 1 for a topographical representation of tau and Aβ binding in individual participants. Age correlated negatively with cortical (*r* = –0.83, *p* < 0.01, or β= –0.02, *p* < 0.001) as well as regional tau load across Braak stage regions: entorhinal cortex non-significant at trend (*r* = –0.56, *p* = 0.06, Fig. 2B), hippocampus (*r* = –0.62, *p* = 0.03), occipital (*r* = –0.77, *p* < 0.01) parietal (*r* = –0.90, *p* < 0.001) and frontal cortices (*r* = –0.60, *p* = 0.04). There was no statistically significant relationship between cortical Aβ SUVr values and age.

### Cognitive test results

Twenty participants completed serial pen and paper (ADAS-Cog at baseline and month 6) and computerized cognitive measures (PAL and SWM CANTAB tests at baseline, month 1, month 3 and month 6). Unsurprisingly, in this mildly demented group, no model showed significant change incognition.

We proceeded to explore the relationship between ADAS-Cog scores (baseline cross-sectional and over time) and cortical tau, entorhinal tau, and cortical Aβ posterior cingulate SUVr values for the 12 participants who underwent both tau and amyloid PET that was done at baseline. In terms of the baseline cross-sectional ADAS-Cog models, there were no main effects of any of the four PET variables, and these models were not modulated by age. In the longitudinal ADAS-Cog models (i.e., baseline ADAS-Cog scores subtracted from 6-month scores) entorhinal tau loading did not exert a main effect (β= –1.4, CI [–19.4, 16.6], *p* = 0.9), but its interaction with age was statistically significant (β= 0.3, CI [–0.04, 0.61], *p* = 0.03). This was due to older patients being disproportionately affected by tau loading (see Fig. 2C). Cortical tau did not exert a main effect in the ADAS-Cog longitudinal model, and this was not modulated by age. Both cortical and posterior cingulate Aβ SUVr levels did not reach significance in respect to the cognitive progression model (Fig. 2D). Age and period since symptom onset did not interact significantly with PET Aβ values in these models.

### CSF analyses

**Fig.3:**
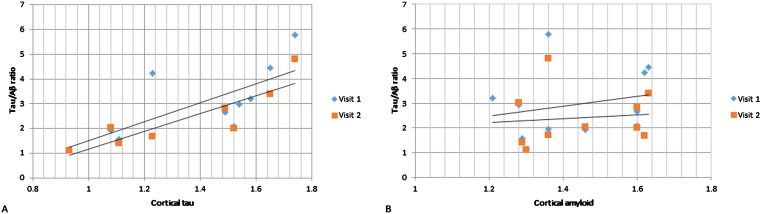
CSF tau/Aβ relationship with PET cortical tau (A) and Aβ (B). On the vertical axes are CSF tau/Aβ ratios and on the horizontal axes are cortical tau (A) and cortical Aβ (B). Visit 1 and Visit 2 CSF measurements in dark gray rombes and light gray rectangles respectively. PET scanning was done at baseline only.

CSF was collected from 16 patients at baseline and from 16 patients at follow-up (13 patients had both baseline and follow-up CSF collection). We found that cortical tau correlated strongly with baseline CSF tau (*r* = 0.80, *p* < 0.01), p-tau (*r* = 0.70, *p* = 0.02) as well as tau/Aβ ratio (*r* = 0.73, *p* = 0.02; Fig. 3) but not Aβ. Cortical Aβ did not correlate with baseline CSF measures (tau, p-tau, Aβ, or tau/Aβ).

In the linear models exploring whether cortical Aβ or tau predict CSF change over the 6-month study period, we found that neither cortical tau nor Aβ were statistically significant in predicting change in tau, p-tau, Aβ, or tau/Aβ over the 6-month period.

To test the hypothesis that CSF markers at baseline predict cognitive decline, we analyzed the relationship between four CSF variables (baseline CSF tau, p-tau, Aβ, and tau/Aβ ratio) to longitudinal cognitive change (ADAS-Cog and PAL tests). Baseline CSF tau, p-tau, and Aβ exerted significant main effects in the ADAS-Cog longitudinal model: tau (β= 0.0095, CI [2.33 0.018] *p* < 0.05), p-tau (β= 0.0974, CI [0.002 0.192] *p* < 0.05), and Aβ (β= 0.0492, CI [0.004 0.081] *p* = 0.03). Tau but not Aβ interacted with age in these models (β= 0.004, CI [4.199 0.001] *p* < 0.05); the interaction in the p-tau model did not reach significance. There was no main effect of baseline tau/Aβ ratio on the ADAS-Cog longitudinal model. There were no main effects of baseline tau, p-tau, Aβ, or tau/Aβ ratio in the PAL longitudinal models.

## DISCUSSION

In this study of people diagnosed with mild AD, we explored the relationships between the recently developed PET tau tracer, [^18^F]AV1451, and established AD research measures: PET Aβ ([^18^F]AV45), CSF measures of tau and Aβ and cognitive measures. We found a relationship between tau and age, whereby younger patients with mild AD had relatively greater tau burden in the entorhinal cortex. Entorhinal cortex was also the only region where PET measures of tau associated with cortical Aβ binding. The relationship between tau and age was underscored by the finding that cognitive deterioration over the 6-month period was associated with tau levels in the entorhinal cortex especially in older participants. Finally, cortical tau correlated strongly with CSF tau measures but there was no relationship between Aβ PET and CSF measures.

### Age effects

The finding of a negative relationship between age and tau deposition appears at odds with evidence for rising MTL tau levels with age [23] but may be reflective of different rates of tau accumulation depending on age of illness onset. A recent tau PET study provided evidence in support of this by showing that while in unimpaired people tau deposition in MTL correlates with age, tau propagation in MCI extends beyond MTL (i.e., inferotemporal and fusiform gyri) in younger and more cognitively impaired individuals [34]. This is consistent with studies showing that in late-onset AD the medial temporal lobes are preferentially affected while younger onset disease features greater cortical degeneration and relative sparing of the hippocampus [8, 35–38].

The age effects observed here may reflect the view that that AD features distinct clinico-pathological phenotypes: a typical, late-onset form affecting preferentially the medial temporal lobe and atypical, younger-onset forms that affect the whole cortex [39]. These younger-onset forms are less dependent on APOE4 risk factors, progress quicker and have a more varied clinical presentation [40, 41]. This form of AD may be a primarily tau-driven disease as in the current study as well as in others there was no relationship between Aβ and age [8]. The relationship we see between age and tau pathology suggests a continuum of disease between young-onset and late-onset disease with the younger group in the present study with an onset in their mid-60 s sharing some of characteristics of young-onset dementia, more usually considered a disorder with an onset before the age of 60.

### Effects of tau and Aβ on change in cognition

Overall, there was no significant deterioration of cognition in our group over the 6-month period, as expected in this very mild group where longer periods are required to observe disease progression. Nonetheless, we found both PET and CSF measures of tau but not amyloid pathology were associated with worsening cognition at 6 months. In the PET models, this association between tau and cognition appeared to be driven primarily by older participants as entorhinal tau levels in the group older than the mean 72.5 y were positively related to cognitive progression, while there was no such relationship in the younger group. The interacting effect of age and tau accumulation on cognitive progression was replicated in the CSF tau models. That tau correlates better with clinical features of dementia than Aβ pathology is in line with other early studies using PET imaging of tau pathology, showing that Braak staging (estimated automatically on the basis of spatial pattern of tau accumulation) correlates with extent of baseline [23, 24] and longitudinal [23] cognitive deficits. We interpret our finding of interaction between tau measures and age on cognition as a suggestion that tau accumulation in the MTL structures such as the entorhinal cortex has a disproportionately negative effect on cognition in older age, i.e., in older people relatively lower levels of tau in the entorhinal cortex are associated with cognitive deterioration. These effects may be due to the lower cognitive reserve in older people or to other pathology contributing to dementia in this age group.

### Relationship between CSF and PET biomarkers

Finally, we compared tau and Aβ PET signal with the best validated marker of AD pathology— tau/Aβ CSF ratio [42]. We found that it was baseline cortical tau but not Aβ that associated with the tau/Aβ ratio. This relationship was due to a correlation between cortical and CSF measures of tau which is in line with the two published studies comparing CSF and PET tau [27, 43]. These results suggest that tau is more closely aligned with pathological processes at the stage of developed illness. This could be due to amyloid reaching a plateau around the time of symptom onset as has been suggested previously [44].

An important difference from existing literature was the lack of a significant negative relationship between cortical Aβ as measured with PET and CSF Aβ [25, 45]. Most studies examining this relationship included both amyloid-positive and amyloid-negative individuals; within either group the correlations are weaker or absent [46]. Our group was relatively homogeneous in terms of PET amyloid signal and therefore the most likely explanation for the negative result was the modest sample size of our cohort and so should be treated with caution.

We also explored the relationship between PET cortical Aβ signal and tau ligand binding in the different regions used for Braak staging. We found that only entorhinal tau correlated with cortical amyloid. This finding could be interpreted as supportive for the ‘trigger and bullet’ hypothesis of the interaction between Aβ and tau in AD pathophysiology [47]— that a critical level of Aβ burden needs to be reached before that toxic tau cascade is triggered starting from the entorhinal cortex. However, the existing evidence suggests that tau accumulation in the MTL is part of normal aging with tau propagating beyond MTL only in those who are classed as Aβ positive [23].

### Strengths and limitations

This is a study with notable strengths and weaknesses. In terms of strengths, it is one of the few studies to report not only both Aβ and tau imaging but also CSF markers of Aβ and tau pathology, and with all measures being conducted within a very short time period. A further strength is the longitudinal (6 month) data on cognition and CSF AD biomarkers. Last but not least, it demonstrated the feasibility of a multicenter, in-depth and high frequency biomarker sampling design which may be crucial for identifying temporal trends in AD biomarker evolution and clinical trials design. In terms of limitations, firstly the study was designed to assess the feasibility and acceptability of the in-depth and frequent biomarker investigations and therefore was not powered for mild to moderate effects sizes. As a result, most of the results should be treated with caution –for instance the lack of replication of a negative relationship between CSF Aβ and tau may be a Type II error on account of the small sample size. Secondly, the study likely suffers from a recruitment bias; the sample consisted of 2–4 patients per site who for the purposes of quick study completion were highly motivated and known to the study investigators and so may not be representative of a randomly selected mild AD group. Thirdly, the study included a modest in length follow-up (6 months), which is likely to be insufficient to answer questions about the longitudinal relationship of AD biomarkers in the mild stages of the disease. Lastly, this study did not include a control group which precluded the possibility for assessing progression data against normative data. We are now in the process of attempting to address the multitude of limitations by setting up a large, multi-site study informed by the acceptability and feasibility currently reported study. In terms of PET methodology, tau tracers are still early in their development and further developments are needed to clarify their reliability and specificity. We note that [^18^F]AV-1451 used in this study has off-target binding of relevance on some neurodegenerative disorders [17], although neuromelanin has been ruled out as a significant determinant of binding in AD [21], and the cortical distribution is unlikely to be explained by monoamineoxidase.

### Conclusion

In this study, we found a significant relationship between age and cortical PET tau whereby younger patients with similar severity of clinical disease have disproportionally more tau pathology. These results argue that younger age onset AD may be primarily driven by tau pathology while AD developing later in life may be driven by a multitude of pathological mechanisms. Nonetheless, cortical Aβ correlated with tau in the entorhinal cortex, the origin of AD pathology which suggests that irrespective of age, AD is a disorder triggered by Aβ deposition and progressed through tau pathology. Critically, in the stage of developed illness cortical tau is a better correlate than Aβ of the best validated AD diagnostic marker— the CSF tau/Aβ ratio. The results of this study remain to be replicated in larger samples.

## DISCLOSURE STATEMENT

Authors’ disclosures available online (http://j-alz.com/manuscript-disclosures/17-0129r1).
